# Defense suppression benefits herbivores that have a monopoly on their feeding site but can backfire within natural communities

**DOI:** 10.1186/s12915-014-0098-9

**Published:** 2014-11-18

**Authors:** Joris J Glas, Juan M Alba, Sauro Simoni, Carlos A Villarroel, Marije Stoops, Bernardus CJ Schimmel, Robert C Schuurink, Maurice W Sabelis, Merijn R Kant

**Affiliations:** Department of Population Biology, Institute for Biodiversity and Ecosystem Dynamics, University of Amsterdam, Science Park 904, 1098 XH Amsterdam, Netherlands; Department of Plant Physiology, Swammerdam Institute for Life Sciences, University of Amsterdam, Science Park 904, 1098 XH Amsterdam, Netherlands; CRA-ABP Consiglio per la Ricerca e la Sperimentazione in Agricoltura - Research, Centre for Agrobiology and Pedology, via Lanciola 12/a, 50125 Florence, Italy

**Keywords:** Plant-herbivore interactions, Plant defense, Phytohormones, Defense suppression, Plant-mediated indirect interactions, Community ecology, *Solanum lycopersicum*, *Aculops lycopersici*, *Tetranychus urticae*, *Pseudomonas syringae*

## Abstract

**Background:**

Plants have inducible defenses to combat attacking organisms. Hence, some herbivores have adapted to suppress these defenses. Suppression of plant defenses has been shown to benefit herbivores by boosting their growth and reproductive performance.

**Results:**

We observed in field-grown tomatoes that spider mites (*Tetranychus urticae*) establish larger colonies on plants already infested with the tomato russet mite (*Aculops lycopersici*). Using laboratory assays, we observed that spider mites have a much higher reproductive performance on russet mite-infested plants, similar to their performance on the jasmonic acid (JA)-biosynthesis mutant *def-1*. Hence, we tested if russet mites suppress JA-responses thereby facilitating spider mites. We found that russet mites manipulate defenses: they induce those mediated by salicylic acid (SA) but suppress those mediated by JA which would otherwise hinder growth. This suppression of JA-defenses occurs downstream of JA-accumulation and is independent from its natural antagonist SA. In contrast, spider mites induced both JA- and SA-responses while plants infested with the two mite species together display strongly reduced JA-responses, yet a doubled SA-response. The spider mite-induced JA-response in the presence of russet mites was restored on transgenic tomatoes unable to accumulate SA (*nahG*), but russet mites alone still did not induce JA-responses on *nahG* plants. Thus, indirect facilitation of spider mites by russet mites depends on the antagonistic action of SA on JA while suppression of JA-defenses by russet mites does not. Furthermore, russet mite-induced SA-responses inhibited secondary infection by *Pseudomonas syringae* (*Pst*) while not affecting the mite itself. Finally, while facilitating spider mites, russet mites experience reduced population growth.

**Conclusions:**

Our results show that the benefits of suppressing plant defenses may diminish within communities with natural competitors. We show that suppression of defenses via the JA-SA antagonism can be a consequence, rather than the cause, of a primary suppression event and that its overall effect is determined by the presence of competing herbivores and the distinct palette of defenses these induce. Thus, whether or not host-defense manipulation improves an herbivore’s fitness depends on interactions with other herbivores via induced-host defenses, implicating bidirectional causation of community structure of herbivores sharing a plant.

**Electronic supplementary material:**

The online version of this article (doi:10.1186/s12915-014-0098-9) contains supplementary material, which is available to authorized users.

## Background

In nature and in agriculture, plants suffer from a diverse community of herbivores and pathogens. Upon attack by these organisms, plants undergo rapid physiological changes which take place not only at the site of attack, but also in the undamaged parts of attacked leaves and in distal undamaged (systemic) leaves, thereby increasing resistance plant-wide [1]. Two hormone signaling pathways play a major role in the regulation of defense responses: the jasmonic acid (JA)-pathway, which is generally induced by herbivores and necrotrophic pathogens [2], and the salicylic acid (SA)-pathway, which orchestrates defenses mainly against biotrophic pathogens [3] as well as phloem-feeding herbivores [4].

The JA and SA defense pathways have often been observed to display negative cross-talk, whereby an increase in the level of one phytohormone reduces the defense responses under control of the other [5] and this is possibly adaptive since it could allow plants to fine-tune the balance between different defensive strategies [6-8]. Yet, some herbivores have adapted to manipulate plant defenses to their own benefit [9-14]. Some of these, such as whiteflies, suppress JA defenses via induction of SA defenses [9,15-17] while others, for instance the red tomato spider mite *Tetranychus evansi*, suppress both JA and SA defenses [11]. Suppression may also occur independent from both the JA and SA pathways as observed for *Spodoptera littoralis* caterpillars [18].

In communities of organisms attacking plants, where different species can differentially activate host plant defenses, induction by one species may also affect the performance of another [13,19-24], implying that apart from directly competing for the same resource (that is, ‘exploitation competition’) herbivores may also indirectly compete with each other via induced changes in the quality of the plant (that is, ‘plant-mediated indirect interactions’) [25]. Thus, although induction or suppression of defenses may demote or promote an herbivore’s reproductive performance when feeding in isolation, the actual fitness benefits may be uncertain within its community since competitors may share the same benefits.

**Figure 1:**
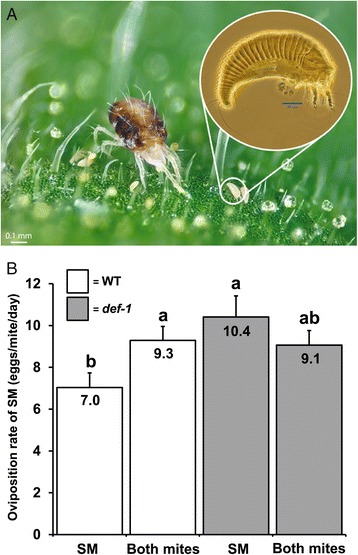
**Spider mites benefit from suppression of JA-defenses by russet mites in WT tomato plants to the same extent as they benefit from the** ***def-1*** **mutation.** An adult female of the two-spotted spider mite (*Tetranychus urticae*) (left) residing together with the tomato russet mite (*Aculops lycopersici*) (right, inset) on a tomato (*S. lycopersicum*) leaflet **(A)**. Average number of eggs produced by spider mites (SM) on wild-type (WT) (cv. CM) and JA-deficient *def-1* plants that were pre-infested with russet mites (Both mites) or not (SM). Values in the bars (+SE) indicate mean oviposition rates and different letters above bars denote significant differences (ANOVA followed by Fisher’s LSD test, *P* <0.05). Three leaflets per plant were analyzed. In total, eight plants per treatment were analyzed **(B)**. ANOVA, analysis of variance; JA, jasmonic acid; LSD, least significant difference.

The two-spotted spider mite (*Tetranychus urticae*) (Acari) and the tomato russet mite (*Aculops lycopersici*) (Acari) are minute tomato pests: the first belongs to the Tetranychidae of which adults reach a body size of around 0.5 mm and the second to the Eriophyidae, the smallest terrestrial animals on earth, of which adults reach a length of about 100 to 140 μm (Figure 1A). We observed in field-grown tomatoes in Italy that tomato russet mites often co-occur with two-spotted spider mites. On such co-infested plants in the field, spider mites reached significantly higher population densities than on plants without russet mites. Based on these observations, we asked the question whether russet mite infestations change the host plant’s physiology in a way that benefits spider mites. Our data show that russet mites suppress JA-defenses and promote the performance of spider mites. However, the latter is not directly caused by the first: both mite species also induce SA defenses and these responses add up when the two species are together, resulting in a second suppression event, that is, that of the spider mite-induced JA-defense response due to the antagonistic effect which the doubled SA response has on this JA-response [5]. In contrast, we found that suppression of JA-defenses by russet mites alone occurs independently from SA. Furthermore, we show that growth of the bacterial pathogen *Pseudomonas syringae* pv. *tomato* DC3000 (*Pst* DC3000) is inhibited by russet mite-induced SA. Finally, we show that russet mite population growth decreases in the presence of spider mites, indicating that the benefits of defense suppression for an herbivore that monopolizes its feeding site can backfire in the presence of competitors.

## Results

### Spider mites reach higher densities on plants with russet mites in the field

Tomatoes growing in the field in Italy were sampled to determine the (co-)occurrence of plant-eating mites and their respective densities earlier and later in the season. Spider mites and/or russet mites were found at 85 sampling sites and in 33 of those sites the two species were found together on the same plant (see Additional file 1: Figure S1). Spider mites reached significantly higher densities on leaflets with than on leaflets without russet mites (see Additional file 2: Table S1 and Figure S2). In contrast, the number of russet mites was reduced on dual-infested leaflets compared to leaflets on which russet mites were feeding alone, albeit not significantly (28.4 ± 8.3 versus 15.4 ± 2.7 mites/leaflet) (see Additional file 2: Table S1).

### Russet mites promote reproductive performance of spider mites on tomato

To test the hypothesis that spider mites benefit from russet mite infestations, plants were infested with russet mites and spider mites and spider mite reproductive performance was assessed by comparing the number of spider mite eggs produced on plants that had been pre-infested with russet mites versus plants that had been infested with spider mites only. Spider mites produced over 25% more eggs on wild-type (WT) plants that had been pre-infested with russet mites as compared to plants infested with spider mites only (Figure 1B). We hypothesized that this effect may be linked to tomato JA-defenses since these constitute a major anti-spider mite defense [26]. Indeed, compared to WT plants, spider mite reproductive performance increased to the same magnitude on the JA-biosynthesis mutant *defenseless-1* (*def-1*) [26] (Figure 1B), while the number of eggs produced was reduced on transgenic *35S::prosystemin* (*prosys+*) plants (see Additional file 3: Figure S3). *Prosys +* plants display a constitutively activated JA-signaling pathway [27], and, hence, this confirms that spider mites are susceptible to JA-dependent defenses [10,28]. In contrast to WT plants, russet mites did not affect spider mite oviposition rate on *def-1* plants (Figure 1B) nor on *prosys +* plants (see Additional file 3: Figure S3), suggesting that suppression of inducible JA defenses by russet mites accounts for the observed effect on spider mite fecundity.

### Russet mites suppress JA-defenses

To test the hypothesis that russet mites suppress JA-defenses in tomato, we measured defense responses in leaflets of spider mite- and russet mite-infested plants by quantifying levels of the phytohormones JA, JA-Ile and SA as well as transcript levels of a few well-established JA- and SA-related defense marker genes. As markers for the JA-pathway we used four genes: (1) *Polyphenol oxidase-F* (*PPO-F*), expressed in tomato leaves and also in type VI glandular trichomes [29], (2) *Threonine Deaminase-II (TD-II)*, involved in resistance against herbivores and tightly regulated by the JA-signaling pathway [30], (3) *Jasmonate-Inducible Protein 21* (*JIP-21*), inducible to high levels by wounding and methyl jasmonate [31] and (4) *Wound-induced Proteinase Inhibitor II* (*WIPI-II*) [32], known to be induced in tomato by spider mites [33]. As a marker for the SA-pathway we selected *Pathogenesis-related Protein 6* (*PR-P6*), which is also induced by spider mite feeding on tomato [33].

**Figure 2:**
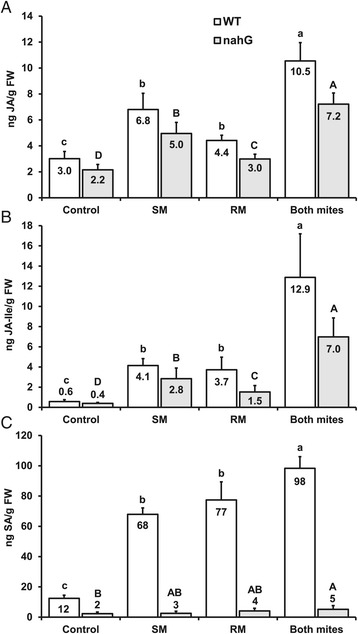
**Phytohormone levels in leaflets infested with spider mites, russet mites, and both species simultaneously.** The amounts of endogenous JA **(A)**, JA-Ile **(B)** and SA **(C)** in wild-type (WT) tomato (cv. MM) and *nahG* leaflets infested with spider mites (SM), russet mites (RM) or both species (Both mites) together (seven days after infestation). Values represent the means (+SE) in nanogram (ng) per gram fresh weight (g FW) of nine to ten plants from two independent experiments. Different lowercase letters denote significant differences for WT plants; uppercase letters denote significant differences for *nahG* plants (ANOVA followed by Fisher’s LSD test; *P* <0.05). ANOVA, analysis of variance; JA, jasmonic acid; LSD, least significant difference; SA, salicylic acid; SE, standard error.

**Figure 3:**
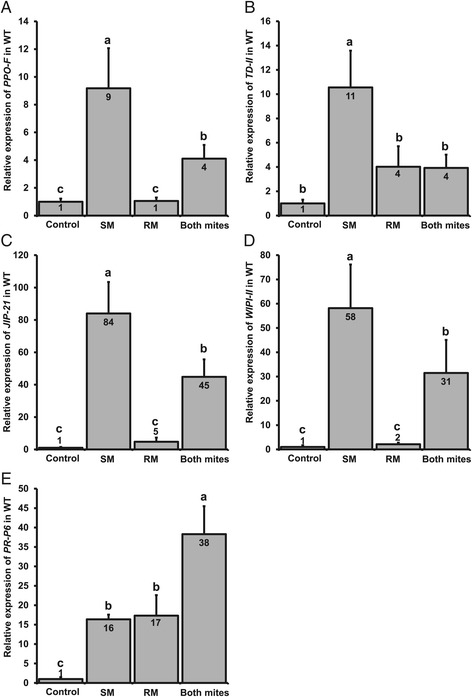
**Russet mites suppress spider mite-induced expression of JA-marker genes but induce expression of a SA-marker gene.** Relative transcript levels of *PPO-F* **(A)**, *TD-II* **(B)**, *JIP-21* **(C)**, *WIPI-II* **(D)** and *PR-P6* **(E)** in wild-type (WT) tomato (cv. MM) leaflets infested with spider mites (SM), russet mites (RM) or both species (Both mites) together (seven days after infestation). Values (+SE) represent the mean of 11 to 13 plants from three independent experiments. Different letters above the bars denote significant differences in expression levels between treatments (ANOVA followed by Fisher’s LSD test; *P* <0.05). ANOVA, analysis of variance; JA, jasmonic acid; LSD, least significant difference; SA, salicylic acid; SE, standard error.

As compared to uninfested control plants, both russet mites and spider mites induced the accumulation of JA and JA-Ile in WT tomato (Figure 2A, B). Strikingly, whereas in spider mite-infested plants expression of the JA-related marker genes was strongly upregulated as well, this did not happen in russet mite-infested plants (Figure 3A-D), despite the upstream phytohormone accumulation. Expression of *PPO-F* was upregulated 9-fold by spider mites but not by russet mites whereas expression of *TD-II*, *JIP-21* and *WIPI-II* was upregulated 11-, 84- and 58-fold above control levels by spider mites respectively, yet only 4-, 5- and 2-fold by russet mites (Figure 3A-D). Furthermore, both russet mites and spider mites induced a strong increase in the levels of SA (Figure 2C) and, accordingly, both species triggered a strong and comparable up-regulation of *PR-P6* (Figure 3E). Hence, even though both mite species induce similar levels of SA, JA and JA-Ile only spider mites upregulate JA-marker genes, indicating that russet mites suppress the downstream JA-defense response.

To test whether the presence of russet mites on plants suffices to down-regulate the spider mite-induced JA-response as well, we assessed defense responses in plants infested with both mite species. We found that JA, JA-Ile and SA accumulated to significantly higher levels in plants infested with both species compared to plants that had been infested with either of the two species alone (Figure 2). However, despite increased accumulation of JA and JA-Ile, transcript levels of all four JA-defense marker genes were suppressed to intermediate levels significantly below the levels in leaflets infested with spider mites only (Figure 3A-D), whereas, in contrast, induction of the SA-marker gene *PR-P6* doubled (Figure 3E). The fact that JA-levels remained high in simultaneously infested plants strengthens the conclusion that suppression occurs downstream from JA-biosynthesis.

### Russet mites suppress JA-defenses independent from SA, but suppression of spider mite-induced JA-defenses depends on SA

Subsequently, considering the significantly increased *PR-P6* expression levels in simultaneously infested plants, we tested if suppression of JA-defenses is mediated via the antagonistic effect of SA on the JA-pathway. For this test, we used tomato plants carrying the *35S::nahG* transgene, which converts endogenous SA into catechol. *nahG* plants are therefore unable to accumulate SA [34].

**Figure 4:**
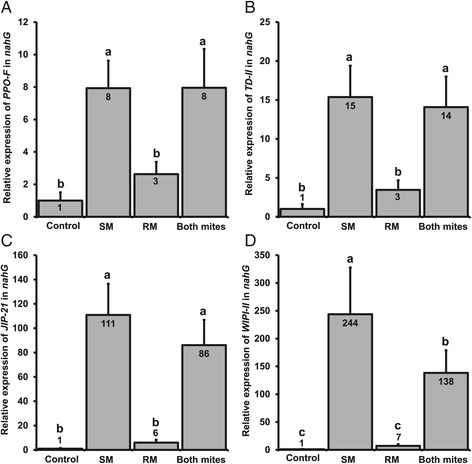
**Russet mite-mediated suppression of the spider mite-induced JA-response depends on SA.** Relative transcript levels of *PPO-F* **(A)**, *TD-II* **(B)**, *JIP-21* **(C)** and *WIPI-II* **(D)** in *nahG* plants infested with spider mites (SM), russet mites (RM) or both species (Both mites) together (seven days after infestation). Values (+SE) represent the mean of 12 to 13 plants from three independent experiments. Different letters above the bars denote significant differences in expression levels between treatments (ANOVA followed by Fisher’s LSD test; *P* <0.05). ANOVA, analysis of variance; JA, jasmonic acid; LSD, least significant difference; SA, salicylic acid; SE, standard error.

As in WT plants, spider mites as well as russet mites induced a significant accumulation of JA and JA-Ile in *nahG* plants (Figure 2A, B). In *nahG* plants, only marginal amounts of SA were detected (Figure 2C), confirming that SA accumulation is effectively blocked due to degradation by the enzyme salicylate hydroxylase. Furthermore, as in WT plants, spider mite feeding caused a strong up-regulation of all four JA-marker genes in *nahG* plants whereas these genes were induced only marginally by russet mites alone and 3 to 35 times less than by spider mites (Figure 4). Expression levels of *PPO-F, TD-II*, *JIP-21* and *WIPI-II* were upregulated respectively 8-, 15-, 111- and 244-fold above control levels by spider mites, yet only 3-, 3-, 6- and 7-fold by russet mites (Figure 4), similar to the pattern observed in WT plants. However, in contrast to WT plants, spider mite-induced expression of three out of the four JA-marker defense genes (that is, *PPO-F*, *TD-II*, *JIP-21*) was restored in *nahG* plants infested with both mite species simultaneously (Figure 4). These results indicate that suppression of JA-responses by russet mites alone and suppression of JA-responses induced by spider mites are two distinct events. Suppression of the spider mite-induced JA-response by russet mites is due to the antagonistic effect of SA on the JA-pathway, whereas suppression of downstream JA-defenses by russet mites is independent from SA.

### Spider mites inhibit population growth of russet mites

**Figure 5:**
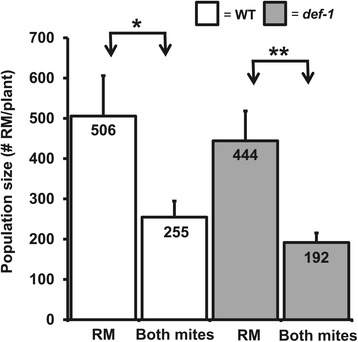
**Spider mites inhibit russet mite population growth.** Russet mite population size (number of mites/plant; + SE) on wild-type (WT) (cv. CM) and *def-1* plants that had been infested with russet mites alone (RM) or co-infested with spider mites (Both mites) 14 days after infestation with RM. Plants had been infested with RM for seven days when SM was introduced. Values represent the mean of 13 to 15 plants. Asterisks represent significant differences as determined by Student *t*-test (*, *P* <0.05; **, *P* <0.01). SE, standard error.

Spider mites displayed an increased reproductive performance on tomato WT leaflets previously infested with russet mites. To determine the consequence of increased spider mite fecundity on russet mite performance, russet mite-infested plants were subsequently infested with spider mites and russet mite population growth was measured as compared to plants infested with russet mites only. After 14 days of infestation, russet mite population size was approximately 50% lower on tomatoes infested with spider mites than on plants without, and a similar effect was observed on *def-1* plants (Figure 5), indicating that russet mites suffer from competition by spider mites.

### Russet mite-induced SA-defense inhibits bacterial growth

**Figure 6:**
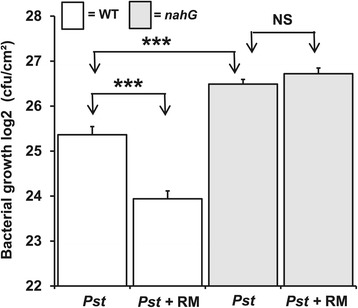
**Russet mite-induced SA inhibits growth of** ***Pst*** **DC3000 on tomato.** *Pst* population growth, quantified as the number of colony forming units (CFU/cm^2^; + SE), in wild-type (WT) (cv. MM) and *nahG* plants that were either without RM (*Pst*) or had been pre-infested with RM (*Pst* + RM) for seven days. In total, seven plants were used per genotype and per treatment. The asterisks indicate a statistically significant difference (Student’s *t*-test; *P* <0.001). RM, russet mites; SA, salicylic acid; SE, standard error.

To assess if the observed induction of russet mite-induced SA-defenses is biologically significant, we assessed its consequence for growth of the SA-sensitive bacterial pathogen *Pst* DC3000 [35]. DC3000 is frequently found on tomatoes in the same rural areas where the two mite species also occur (see Additional file 4: Figure S4). Bacterial growth was strongly reduced in leaflets of WT plants that had been pre-infested with russet mites compared to uninfested control leaflets, whereas, on *nahG* plants the presence of russet mites did not affect *Pst* growth (Figure 6). Hence, this result indicates that russet mites increase resistance of tomatoes to *Pst* and also that *Pst* is susceptible to russet mite-induced SA.

To assess whether reduced bacterial performance would affect russet mite performance, we then infiltrated russet mite-infested plants with *Pst* and measured mite population growth on WT and *nahG* plants. *nahG* plants were included as a control treatment, since on these plants russet mite infestations did not interfere with *Pst* growth (Figure 6). *Pst* infections did not have a significant effect on russet mite population growth, neither on WT nor on *nahG* plants (see Additional file 5: Figure S5), indicating that the effect of russet mites on *Pst* growth cannot be explained by differences in russet mite density. Moreover, since russet mite population growth was similar on WT and *nahG* plants (see Additional file 5: Figure S5), it is clear that russet mites are not affected by the SA-mediated defenses they induce in WT plants.

### Russet mites display a faster growth rate on a JA-defense mutant

Russet mites facilitate spider mites, but interfere with the population growth of *Pst*, indicating that there are considerable ecological costs and benefits associated with induction and suppression of plant defenses (see Additional file 6: Figure S6). To test if we could demonstrate the costs associated with defense suppression by russet mites we subsequently allowed russet mites to build up a population on JA-deficient *def-1* plants in comparison to WT plants. This experiment suggests that suppressing defenses is costly since after 16 days of infestation (equivalent to two generation cycles) russet mite populations had grown larger on *def-1* than on WT tomatoes (see Additional file 7: Figure S7).

## Discussion

By combining data on tomato defense-gene expression and phytohormone levels we have dissected the relative contribution of JA- and SA-defense responses to the reproductive performance of spider mites and russet mites alone as well as when sharing a host plant. Even though both russet mites and spider mites induced the accumulation of JA and its active form JA-Ile [36] in tomato leaflets (Figure 2A, B), we found that feeding by russet mites did not, or only marginally, upregulate the expression of JA-defense marker genes, whereas spider mites upregulated these genes to much higher levels (Figure 3A-D). From this result, we conclude that russet mites suppress the JA-mediated downstream defense response. In contrast, both spider mites and russet mites induced accumulation of SA (Figure 2C) and expression of the SA-defense marker gene *PR-P6* (Figure 3E). Hence, although the two mite species feed from different tomato cell types, that is, russet mites from epidermal cells and spider mites from parenchyma cells [37,38], they induce tomato SA-defenses similarly.

In agreement with previous studies, spider mite feeding resulted in a strong JA-defense response [28,33], as evidenced by a significant increase in JA-Ile accumulation (Figure 2B) and in JA-marker gene expression (Figure 3A-D). However, in simultaneously infested plants the expression of JA-defense marker genes was reduced to levels significantly below the levels in leaflets infested with spider mites only (Figure 3A-D), while *PR-P6* transcript levels doubled in the presence of both mite species (Figure 3E). Therefore, considering the well-documented antagonistic cross-talk between JA and SA [5], we subsequently tested if suppression of JA-marker genes by russet mites could be attributed to the antagonistic interaction of SA onto JA. We observed that spider mite-induced JA-marker gene expression was restored in SA-deficient *nahG* plants that had been simultaneously infested with russet mites (Figure 4A-C), suggesting that, indeed, suppression of spider mite-induced JA-defenses is due to negative cross-talk with SA. However, russet mites alone still suppressed JA-responses in *nahG* plants (Figure 4). Therefore, we conclude that in leaflets infested with both mite species cross-talk is a consequence, and not the cause, of the primary suppression of JA-defenses by russet mites and that in plants infested with both species their induced SA-responses add up and antagonize the spider mite-induced JA-response as a secondary effect. These results also suggest that suppression of JA-responses by russet mites occurs fairly locally near feeding sites and does not spread throughout the whole leaflet. We did not find evidence for cross-talk at the phytohormone level as absence of SA did not lead to an extra increase in spider mite-induced JA or JA-Ile [39]. In fact, levels of JA-related phytohormones tended to be lower in *nahG* plants compared to WT plants (Figure 2A, B).

Interestingly, the intermediate expression of *WIPI-II* in simultaneously-infested WT plants (Figure 3D) was not restored in *nahG*. This suggests that *JIP-21*, *PPO-F* and *TD-II* are antagonized differently than *WIPI-II* by russet mites. Since *WIPI-II* expression depends negatively on NONEXPRESSOR OF PATHOGENESIS-RELATED PROTEINS1 (NPR1) [40], other hormones, for example, SA-derivatives [41], may modulate NPR1 as well or be co-regulated by NPR1-independent processes [42].

Most notably, spider mites induced SA themselves to similar levels as russet mites. Since spider mites induce JA- and SA-responses simultaneously (Figures 2 and 3) this raises an intriguing question: why does the russet mite-induced SA-response suppress spider mite-induced JA defenses but the SA induced by spider mites alone does not? We hypothesize that actually spider mite-induced SA-responses also antagonize simultaneously induced JA responses, albeit to intermediate induction levels while only the total SA-response induced by the two mites together is powerful enough to suppress these intermediate levels to levels significantly below those in plants infested with spider mites only. This hypothesis is supported by the spider mite-induced JA-marker gene expression data in *nahG*: the relative expression of three of the four JA-defense markers was much stronger in *nahG* compared to WT plants, that is, relative expression of *TD-II*, *JIP-21* and *WIPI-II* was, respectively, about 40%, 30% and 400% higher in *nahG* plants than in WT plants (compare Figure 3B-D versus Figure 4B-D), suggesting that the spider mite-induced JA-response may be partially suppressed by the simultaneously induced SA-response in WT plants. If so, induction of SA-responses by spider mites could be adaptive. However, this suppression does not increase with increasing mite densities since the levels of both hormones, and their downstream responses, increase similarly when more SM are feeding [43].

The fact that suppression of defenses affects responses induced by a competing species begs the question if such indirect interactions can influence an herbivore’s community structure. Cultivated tomato can be attacked by as many as 100 to 200 different arthropod herbivores [44], and russet mites are a worldwide pest of tomatoes [45]. Considering this, it is likely that, in field grown crops as well as in greenhouses, russet mites often co-occur with other pest species. Hence, we investigated the consequences of russet mite-induced defenses for two naturally co-occurring pest species of russet mites: the two-spotted spider mite *T. urticae* and the bacterial phytopathogen *Pst* DC3000. As shown here, spider mites had a significantly higher reproductive performance in the presence of russet mites, whereas growth of *Pst* was inhibited. This may explain why russet mites induce SA-defenses: it may limit the occurrence of secondary infections by pathogens. However, since *Pst* did not limit russet mite growth (see Additional file 5: Figure S5) we did not find direct evidence that SA-induction is adaptive for russet mites. Yet, since *Pst-*infected plants probably will die earlier, suppressing it could be beneficial for the mite. Russet mite population growth did not differ between WT and *nahG* plants indicating that they are not affected by the SA-defenses they induce (see Additional file 5: Figure S5). Since russet mites suppress JA-defenses independent from SA this finding was in line with our expectation. However, *nahG* mutants suffer from catechol-related side-effects [46,47] and, therefore, confirmation using an independent tomato SA-mutant would be desirable.

One may wonder to what extent the presence of russet mites on a spider mite-infested plant also affects the performance of these spider mites via direct interference. If so, reduced JA-defenses could be due to a reduced spider mite feeding intensity. However, several data contradict this direct interference hypothesis. First, we observed that spider mites reached higher densities on russet mite-infested plants in the field indicating facilitation (see Additional file 2: Supplemental text). Second, in the lab we observed that spider mites laid more eggs on WT tomato plants that had been pre-infested with russet mites as compared to uninfested plants (Figure 1B), suggesting their food intake increased rather than decreased. Third, expression of *PR-P6* was doubled in WT plants simultaneously infested with both species compared to plants infested with either of the species alone, also suggesting that their feeding intensity increased rather than decreased. Fourth, for three of the four JA-marker genes suppression was not observed in simultaneously infested *nahG* plants, which implies that spider mites keep on eating and damaging these plants, also in the presence of russet mites. Hence, plant-mediated factors must have been the primary cause for the observed effects. Notably, russet mites densities in greenhouses have been reported to be much higher than those used in the present study [48]. Therefore, it is well possible that under such conditions the intermediate responses we have observed during co-infestation may escalate to full suppression, thereby boosting the performance of spider mites even more. Previous studies have shown that induction of the JA and/or SA pathway may decrease [6,22] or increase [22,49] the performance of competing organisms on the same host. In addition, a few studies, on spider mites, whiteflies and aphids, have examined the effects of defense suppression on other species. JA-defense suppressing genotypes of the spider mite (*T. urticae*) were found to have a significant positive effect on the performance of ‘inducer’ *T. urticae* genotypes (that is, mite genotypes that normally induce the JA- and SA-pathway) when sharing the feeding site with ‘suppressor’ genotypes [10]. Also, *T. urticae* laid more eggs on leaf discs on which the JA-defense suppressing spider mite *T. evansi* had fed previously, whereas feeding by the inducer species *T. urticae* resulted in decreased oviposition by *T. evansi* [49]. Similarly, the cabbage aphid (*Brevicoryne brassicae*) inhibits JA-accumulation, and this correlated with increased growth and development of caterpillars of the large cabbage white butterfly (*Pieris brassicae*) [13], and, finally, suppression of JA-defenses by whiteflies (*Bemisia tabaci*) improved the performance of spider mites on lima bean (*Phaseolus lunatus*) [50]. Notably, in the whitefly-spider mite [50], as well as in the aphid-caterpillar example [13], suppression of JA defenses was shown to be independent from SA, even though the latter was induced by whiteflies [50].

## Conclusions

Our results highlight the central role of plant-mediated indirect interactions in shaping herbivore communities [25]. The results of our study show that crosstalk between the SA and JA pathways plays an essential role in the spider mite-russet mite interaction, despite the observation that russet mites suppress JA-defenses independent from SA. This suggests that russet mites secrete effector molecules into their host plant to target the JA-signaling pathway directly. Moreover, our data show that the fitness benefits of defense suppression depend on the community structure and that such suppression may backfire when competitors take advantage of it as well. Our results imply that the outcome of interactions between multiple herbivores on a shared host can be unpredictable and counter-intuitive since they depend on simultaneous reciprocal changes between individual community members. Also, these interactions can be bidirectional since one herbivore population may respond to plant defenses induced or suppressed by another and, subsequently, also vice versa. Although we did not test all permutations, it is to be expected that the time point, sequence of arrival as well as infestation density of all attackers present on a plant play a major role in determining the strength of these interactions. In summary, we show that herbivore-plant-herbivore interactions are subject to bidirectional causality in that plant herbivores induce plant responses that may positively or negatively impact them and their competitors, implying that ultimately an herbivore’s fitness depends on how they themselves indirectly alter interactions within their community. We believe these findings have major implications for understanding under which conditions induction and suppression of plant defenses can be adaptive.

## Methods

### Plants, mites and bacteria

Tomato seeds (*Solanum lycopersicum* cv. Castlemart (CM), *defenseless-1* (*def-1*), and a transgenic line *35S::prosystemin* (*prosys+*), both in the genetic background of CM), as well as *S. lycopersicum* cv. Moneymaker (MM) and the transgenic line *nahG* (in the genetic background of MM) were germinated in soil and grown in a greenhouse compartment at a temperature of 25°C and a 15/9 hour light/dark regime. One week prior to each experiment, plants were transferred to a climate room with day/night temperatures of 27ºC/25ºC, a 16/8 h light/dark regime and 60% relative humidity (RH). *Def-1* plants are deficient in wound- and systemin-induced JA accumulation and in the expression of downstream defense genes [26]. *Prosys +* plants overexpress the prosystemin gene, resulting in a constitutively activated JA-signaling pathway [27]. *nahG* plants are transformed with the bacterial gene *nahG* encoding salicylate hydroxylase which removes endogenous SA by converting it into catechol [34]. Both the *prosys +* and the *nahG* gene are under the control of the constitutively expressed CaMV 35S promoter. Experiments with WT and mutant/transgenic lines were always carried out in parallel.

Tomato russet mites (*Aculops lycopersici*, also referred to as russet mites or abbreviated as RM) (Acari: Eriophyidae) were obtained from Koppert Biological Systems (Berkel en Rodenrijs, the Netherlands), who, in turn, had obtained them in the summer of 2008 from naturally infested plants in a greenhouse in the Westland area (the Netherlands), and were since then reared in insect cages (BugDorm-44590DH, Bug Dorm Store, MegaView Science Co., Taichung, Taiwan) in a climate room on tomato plants (cv. CM) that were between three and five weeks old.

Two-spotted spider mites (*T. urticae*, also called spider mites or abbreviated as SM) (Acari: Tetranychidae) were originally obtained in 2001 from a single European spindle tree (*Euonymus europea L.*), in the dunes near Santpoort, the Netherlands (GPS coordinates: 52 26.503 N, 4 36.315 E). The strain we used has been described as a JA-inducing mite genotype and as susceptible to these defenses [10]. Since its collection from the field, the strain has been propagated on detached bean (*Phaseolus vulgaris*) (cv. 746 Speedy) leaves that were placed with the abaxial surface on wet cotton wool and maintained in a climate room (temperature of 25°C, a 16/8 hour light/dark regime and 60% RH).

*Pseudomonas syringae* pv. *tomato* DC3000 bacteria were grown on King’s Broth (KB) medium [51] agar plates, containing rifampicin (50 μg/ml), and grown at 28°C for two to three days. Subsequently, single colonies were picked and bacterial cultures were grown overnight at 28°C in liquid KB medium with rifampicin (50 μg/ml). Bacterial cells were collected by centrifugation (3,000 rpm for 10 minutes), resuspended in 10 mM MgSO_4_ and adjusted to the required optical density (OD) before pressure infiltration into the leaflets.

### Infestation and sampling of plants used for gene expression and phytohormone analyses

At the start of the experiments, 21-day-old tomato plants were infested with spider mites (SM), russet mites (RM) or with the two species together. RM infestations were done by transferring the mites on small pieces of leaflets (about 0.5 cm^2^) to the leaflets of uninfested plants. These leaflet pieces had been cut from leaves picked from a well-infested tomato plant and each piece contained about 250 mobile stages of RM as determined with a stereomicroscope. Plants with spider mites received five spider mites per leaflet on each of three leaflets per plant. Thus, each plant received 15 spider mites in total. RM and SM were introduced at the same time. To prevent mites from dispersing we applied a thin barrier of lanolin (Sigma-Aldrich Chemie B.V., Zwijndrecht, The Netherlands) on the petioles of leaflets that were chosen for infestation. Uninfested control plants received lanolin, but no mites. In total, three leaflets per plant were infested which were pooled at the time of sampling. This sample was taken as one biological replicate. Sampled leaflets were flash-frozen in liquid nitrogen and stored at −80°C until total RNA or phytohormones were extracted. We always picked the same leaflets for infestation, that is, one leaflet of the second compound leaf (counted from the bottom to the top of the plant), one from the third compound leaf and the terminal leaflet of the fourth compound leaf. Sampling was performed seven days after infestation. Generally, starting with a density of about 250 RM per leaflet infection symptoms became visible five to six days after infestation. After seven days of infestation (the time-point of sampling) symptoms of RM-infestation were clearly visible, but leaflets were not necrotic or senesced. The relative transcript levels presented in Figures 3 and 4 represent the mean of 11 to 13 biological replicates, obtained from three independent experiments. The phytohormone levels presented in Figure 2 represent the mean of nine to ten biological replicates, obtained from two independent experiments.

### Sampling for mites in tomato fields

Tomatoes growing in the field were sampled to determine the (co-)occurrence of plant-eating mites. Samplings were performed in several areas of Italy that are well-known for their tomato production (see Additional file 1: Figure S1). Samplings were performed at 93 different sites in total, in the summers of 1997 and 1998. On each site at least two samples were taken, the first one in July and the second one in September.

The sampling method we employed is commonly used for estimating mite densities in a wide variety of horticultural crops in agricultural fields [52]. Briefly, one leaf (that is, a compound leaf) was picked per plant by walking along the tomato rows. Plants were vertically subdivided in three parts: basal, mid and apical. One-third of the total number of leaves collected at a particular site consisted of leaves from the basal part, one-third from the central part and one-third from the apical part of the plants. Leaves were collected from plants from at least 15 to 18 different rows in each field and plants from which leaves were collected were standing at least one meter (2 to 3 plants) from each other. In total, 75 to 100 leaves were collected for each sample. Subsequently, these leaves were examined under a stereomicroscope and on each leaflet the presence/absence of spider mites as well as that of russet mites was recorded. Numbers of spider mites and russet mites present were counted.

### Quantification of gene expression by qRT-PCR

Leaflets were cut at the base and three leaflets per plant were pooled in 50-ml tubes, flash frozen in liquid nitrogen, and stored at −80°C. Leaflets were ground in liquid nitrogen, and total RNA was extracted using a phenol-LiCl-based method as described [53]. The integrity of RNA was checked on 1% agarose gels and subsequently quantified using a NanoDrop 100 spectrophotometer (Fisher Scientific, Loughborough, UK). DNA was removed with DNAse (Ambion, Huntingdon, UK) according to the manufacturer’s instructions, after which a control PCR was carried out to confirm the absence of genomic contaminations. cDNA was synthesized from 2 μg total RNA using a poly(dT) primer and M-MuLV Reverse Transcriptase (Fermentas, St. Leon-Rot, Germany) according to the manufacturer’s instructions. cDNA dilutions (10x) were used as the template in quantitative reverse-transcriptase PCR (qRT-PCR). Reactions were carried out in a total volume of 20 μl containing 0.25 μM of each primer, 0.1 μl ROX reference dye and 1 μl of cDNA template. Two technical replicates were performed per measurement. qRT-PCR was performed with Platinum SYBR Green qPCRSuperMix (Invitrogen, Paisley, UK) using an ABI 7500 (Applied Biosystems, Foster City, CA, USA) system. The program was set to 2 minutes at 50°C, 10 minutes at 95°C, 45 cycles of 15 seconds at 95°C and 1 minute at 60°C, followed by a melting curve analysis. Target gene expression levels were normalized to those of actin. The normalized expression (NE) data were calculated by the ΔCt method NE = (1/(PE_target_^Ct_target^))/(1/(PE_reference_^Ct_reference^) (PE = primer efficiency; Ct = cycle threshold). The PEs were determined by fitting a linear regression line on the Ct-values of a standard cDNA dilution series. Specific amplification was ensured by melting curve analyses and generated amplicons were sequenced. For presenting the qRT-PCR data in the graphs we projected the data on a relative scale by dividing all values by the lowest average value (such that the lowest average is always 1). The primers we used are listed in Additional file 8: Table S2.

### Quantification of phytohormones by means of liquid chromatography-mass spectrometry

Phytohormones were extracted by homogenizing frozen leaf material (approximately 250 mg) in screw cap tubes containing 1 ml of ethyl acetate spiked with 100 ng of D_6_-SA and D_5_-JA (C/D/N Isotopes Inc., Pointe-Claire, Quebec, Canada) as internal standards. Samples were ground twice, using a GenoGrinder (Precellys24 Tissue Homogenizer, Bertin Technologies, Aix-en-Provence, France), at 6,500 rpm for 45 seconds and centrifuged at 13,000 rpm (8 g) for 20 minutes at 4°C. Supernatants from two extraction steps were pooled and evaporated until dryness in a vacuum concentrator (CentriVap Centrifugal Concentrator, Labconco, Kansas City, MO, USA) at 30°C. The dried residue was dissolved in 250 μl 70% methanol, vortexed and centrifuged and the supernatant was transferred to liquid chromatography-mass spectrometry (LC-MS) vials (Fisher Scientific, Hampton, NH, USA). Phytohormone measurements were conducted on a liquid chromatography tandem mass spectrometry system (Varian 320 Triple Quad LC/MS/MS, Agilent Technologies, Santa Clara, CA, USA). Twenty microliters of each sample was injected onto a Pursuit 5 column (C18; 50x2.0 mm). The mobile phase comprised solvent A (0.05% formic acid in water; Sigma-Aldrich, Zwijndrecht, the Netherlands) and solvent B (0.05% formic acid in methanol; Sigma-Aldrich). The program was set as follows: 95% solvent A for 1 minute 30 seconds (flow rate 0.4 ml/minute), followed by 6 minutes in which solvent B increased till 98% (0.2 ml/min) which continued for 2 minutes 30 seconds at the same flow rate, followed by 1 minute 30 seconds at an increased flow rate (0.4 ml/min), subsequently returning to 95% solvent A for 1 minute until the end of the run. Compounds were detected in the electrospray ionization-negative mode. Molecular ions [M-H]^−^ at *m/z* 137 and 209 and 141 and 213 generated from endogenous SA and JA and their internal standards, respectively, were fragmented under 12 V collision energy. The ratios of ion intensities of their respective daughter ions, *m/z* 93 and 97 and *m/z* 59 and 61, were used to quantify endogenous SA and JA, respectively. A standard dilution series of pure compounds of JA-Ile (OlChemIm Ltd, Olomouc, Czech Republic), JA and SA (DUCHEFA Biochemie B.V., Haarlem, the Netherlands) was used to estimate the phytohormone concentrations and the retention time. The amounts were corrected for losses occurring during the extraction with a recovery rate, using the JA and SA internal standards.

### Spider mite reproductive performance assays

To obtain RM-infested leaflets before the start of the SM-performance experiments, 21-day-old tomato plants were infested with RMs as described earlier.

Subsequently, seven days after plants had been infested with RM, four young adult female SM were placed on the adaxial surface of the RM-infested leaflets and on leaflets of the same age and position of non-infested control plants, using a soft bristle paintbrush. After four days (that is, eleven days after the start of the experiment), infested leaflets were detached and SM adults and their eggs were counted using a stereomicroscope. The results presented in Figure 1B represent the mean number of eggs per mite per day of 24 leaflets that were obtained from 8 plants.

In order to obtain SM of the same age, egg waves were generated in a climate room (temperature of 25°C, a 16/8 hours light/dark regime and 60% RH), as previously described [33]. In short, 20 to 30 random adult female spider mites were selected from a rearing colony and allowed to produce eggs for a period of 48 hours on detached bean leaflets on wet cotton wool. After this period, the adults were removed but the eggs were maintained. After 14 days, the 2 ± 2 days young adult females were collected and these were used for the oviposition assays.

### *Pst* DC3000 growth assays

At the start of the assays to assess *Pst* growth (Figure 6), 21-day-old plants were infested with RM as described earlier. Experiments were performed on WT (cv. MM) and SA-deficient *nahG* plants. After seven days of infestation with RM, the left halves (bordering the midrib) of RM-infested leaflets and similar leaflets from non-infested control plants were pressure infiltrated with *Pst* DC3000 (OD_600_ = 0.001) in 10 mM MgSO_4_ using blunt 1 ml syringes (BD Plastipak, Franklin Lakes, NJ, USA). Leaflets from the third compound leaf (counted from the bottom of the plant) were infected. In total, seven plants were infected per treatment and the experiment was repeated a second time with a similar result (see Additional file 9: Figure S8).

Three days after infection with *Pst*, leaflets were detached and two 1-cm^2^ circular leaf discs were punched out from the infiltrated leaflet halves and ground in 500 μl 10 mM MgSO_4_. Serial dilutions were prepared by taking 20 μl of the leaf disc solution and diluting it in 180 μl 10 mM MgSO_4_. Twenty μl of each serial dilution was plated on KB medium + rifampicin (50 μg/mL) plates. The number of colony forming units (CFU) was counted two days after incubation at room temperature.

### Russet mite population growth experiments

For the RM population growth experiments, 21-day-old tomato plants were infested by transferring 20 RM to each of three leaflets per plant. Thus, each plant was infested with 60 RM in total. To prevent mites from dispersing we applied a thin barrier of lanolin on the petioles of leaflets that were chosen for infestation. Uninfested control plants received lanolin, but no mites. Leaflets with a similar position as those used for the gene expression and phytohormone measurements were used for infection. To assess RM performance, infested leaflets were detached and mites (all stages) were washed off by rinsing the leaflets one by one for 20 seconds in 25 ml 100% ethanol. Infested leaflets that came from the same plant were washed in the same solution. RM were counted by running 2 ml of the leaf washes through a particle counting system (PAMAS SVSS, PAMAS, Rutesheim, Germany). Leaf washes were counted 20 seconds after mixing them, to avoid having air bubbles enter the system. Adult RM are around 120 to 150 μm in size while their eggs are around 20 μm. Therefore, the number of particles measured in the range of 50 to 200 μm was used to quantify the number of adult mites. The number of mites per plant was calculated by multiplying the mean number of particles per ml with the total volume of 25 ml. This counting method was validated by means of a dose–response experiment. In this experiment, we infested leaflets on intact plants with 2, 4, 8, 16, 32 and 64 mites per leaflet on each of three leaflets per plant and after 14 days the mites were washed off, as described earlier. The numbers counted implied exponential population growth, corresponding to the starting conditions (see Additional file 10: Figure S9).

For the RM-SM co-infestation experiment (Figure 5), plants were infested by transferring 20 RM to each of the three leaflets per plant. After seven days of RM infestation, half of the plants were subsequently infested with five adult SM on the RM-infested leaflets. Thus, dual-infested plants were infested in total with 15 SM per plant. The population growth of RM was assessed after fourteen days of infestation with RM (and hence after seven days of infestation with SM) by counting the number of RM as described above. The values presented in Figure 5 represent the mean of 13 to 15 plants, obtained in two independent experiments.

For the RM-*Pst* co-infestation experiment (see Additional file 5: Figure S5), plants were infested by transferring 20 RM to each of three leaflets per plant. After seven days of RM infestation, three leaflets of half of the RM-infested plants were infiltrated with *Pst* in 10 mM MgSO_4_ and three leaflets of the other half of the RM-infested plants were infiltrated with mock 10 mM MgSO_4_. The same three leaflets were chosen for infiltration from each plant. Since older leaflets are more susceptible to *Pst* compared to the younger leaflets (JJ Glas, personal observation), the leaflets from the third and fourth compound leaf were infiltrated with a bacterial suspension which had an OD (OD_600_) of 0.0001 while the oldest leaflet (that is, the leaflet on the second fully expanded leaf) was infiltrated with a lower OD_600_ of 0.00005. On each leaflet, approximately ¼ of the leaf area was infiltrated with bacteria. The population growth of RM was assessed after 14 days of infestation with RM (and hence after seven days of infestation with *Pst*) by counting the number of RM as described above. The values presented in Additional file 5: Figure S5 represent the mean of 15 plants, obtained from two independent experiments. Symptoms of *Pst* infection were visible at the time of sampling, with sometimes (minor) parts of the leaflet being senesced and/or necrotic (see Additional file 5: Figure S5).

For assessing RM performance on WT and *def-1* plants (see Additional file 7: Figure S7), plants were infested by transferring 20 RM to each of the three leaflets per plant. RM population growth was assessed after 8, 12 and 16 days by counting the number of RM as described above. The values presented in Additional file 7: Figure S7 represent the means of five to ten plants per time-point, obtained from two independent experiments.

### Statistical analyses

Gene expression data were statistically analyzed using a nested analysis of variance (ANOVA). NE values were compared among treatments using ‘Treatment’ (with the levels ‘Control’, ‘SM’, ‘RM’ or ‘Both mites’) as fixed factor and with the factors ‘Experimental replicate’, ‘Biological replicate’ and ‘Technical replicate’ included as random factors in the model. The factor ‘Technical replicate’ (with levels 1 and 2) was nested to the factor ‘Biological replicate’. The means of each group were compared using Fisher’s LSD (least significant difference) *post hoc* test.

Spider mite oviposition data (Figure 1B; Additional file 3: Figure S3) were analyzed with ANOVA using ‘Treatment’ as fixed factor and including ‘Plant replicate’ as random factor. Means of each group were compared using Fisher’s LSD *post hoc* test.

Phytohormone data (Figure 2) were log-transformed and analyzed using ANOVA, with ‘Treatment’ as fixed factor and ‘Experimental Replicate’ included as random factor in the model. Means of each group were compared using Fisher’s LSD *post hoc* test.

*Pst* performance data (Figure 6; Additional file 9: Figure S8) were analyzed with the Student’s *t*-test. Russet mite population growth data (Figure 5, Additional file 5: Figure S5 and Additional file 7: Figure S7) were log-transformed before statistical analysis. Mite density data (Additional file 2) were log-transformed and analyzed with the Student’s *t*-test for both sampling points independently. Values presented in the graphs represent untransformed data.

Two-sided Student’s *t*-tests were performed in Excel (Microsoft Corporation, Redmond, WA, USA) and ANOVA followed by Fisher’s LSD tests in SPSS 20.0 (SPSS Inc., Chicago, IL, USA).

### Availability of supporting data

Raw data can be accessed via: 10.6084/m9.figshare.1232104.

## Additional files

Additional file 1: Figure S1. Map of Italy with the regions indicated where russet mites and spider mites were found. The color of the symbols indicate which species were found (russet mites = yellow; spider mites = green; both mites = red). Additional file 2:
**Supplemental text Table S1.** Spider mites and russet mites co-occur in the field. **Figure S2.** Spider mites reach higher densities in the presence of russet mites in the field. Spider mite (SM) densities (motile stages/leaflet) were similar at the time of the first sampling but in the second sampling they were higher in the presence of russet mites (Both mites) (Student’s *t*-test on log-transformed numbers; *P* = 0.73 and *P* <0.001, respectively). NS = not significant; the asterisk indicates a statistically significant difference. Russet mite densities (not shown in the figure) were similar at the time of the first sampling but were reduced in the presence of spider mites in the second sampling, albeit not significantly (Student’s *t*-test on log-transformed numbers; *P* = 0.89 and *P* = 0.09). Data are the same as those reported in Table S1. Additional file 3: Figure S3. Spider mite performance is not affected by the presence of russet mites on transgenic *35S::prosystemin* tomatoes. Average number of eggs produced by spider mites (SM) on wild-type (cv. CM) and transgenic *35S::prosystemin* plants (*prosys+*) that were pre-infested with RM (Both mites) or not (SM). Values in the bars (+SEM) represent means and different letters above bars indicate significant differences in SM oviposition rate (ANOVA followed by Fisher’s LSD test, *P* <0.05). Three leaflets per plant were analyzed. In total, 10 plants per genotype and per treatment were analyzed. Additional file 4: Figure S4. Pest distribution data for russet mites (*Aculops lycopersici*), spider mites (*Tetranychus urticae*) and the bacterial pathogen *Pseudomonas syringae* pv. *tomato* (source: [54], accessed on 24/09/2014 and [55]). Additional file 5: Figure S5.
*Pst* DC3000 infections do not interfere with russet mite population growth. Symptoms of bacterial infection on leaflets of wild-type (cv. MM) (A) and *nahG* (B) plants. Russet mite population size (number of mites/plant; + SE) on wild-type (cv. MM) and *nahG* plants infested with RM alone (RM) or co-infected with *Pst* (RM + *Pst*) 14 days after infestation with RM. Plants were infiltrated with *Pst* seven days after infestation with RM. Values represent the mean of 15 plants. Letters above bars indicate results according to ANOVA (*P* = 0.83). Additional file 6: Figure S6. Schematic diagram of the indirect interactions occurring between russet mites, spider mites and *Pst* DC3000. SM = spider mite; RM = russet mite; *Pst* = *Pseudomonas syringae* pv. *tomato*; JA = jasmonic acid; SA = salicylic acid. Additional file 7: Figure S7. Russet mites grow faster on the JA-deficient mutant *def-1* than on wild-type (WT) plants. Values (±SE) represent the mean number of russet mites (RM) per plant, obtained from five to ten plants in two independent experiments. The asterisk indicates a significant difference after 16 days (Student’s *t*-test on log-transformed data; *P* = 0.036). Additional file 8: Table S2. Accession numbers of the transcripts quantified by qRT-PCR and their primer sequences. Additional file 9: Figure S8. Repetition of the *Pst* DC3000 growth experiment. *Pst* population growth, quantified as the number of colony forming units (CFU/cm^2^; + SE), in wild-type (WT) (cv. MM) and *nahG* plants that were either without RM (*Pst*) or had been pre-infested with RM (*Pst* + RM) for seven days. In total, six plants were used per genotype and per treatment. The asterisk indicates a statistically significant difference (Student’s *t*-test; *P* <0.05). Additional file 10: Figure S9. Dose–response experiment. Values (±SE) represent the mean number of russet mites (RM) per plant 14 days after infestation. In total, two plants per density were analyzed.
