# Supplementary material for: Defense suppression benefits herbivores that have a monopoly on their feeding site but can backfire within natural communities
Source: BMC Biol. 2014 Nov 18;12:98. doi: 10.1186/s12915-014-0098-9 (PMC4258945; doi:10.1186/s12915-014-0098-9)
Supplement: Additional file 2: — Supplemental text Table S1. Spider mites and russet mites co-occur in the field. Figure S2. Spider mites reach higher densities in the presence of russet mites in the field. Spider mite (SM) densities (motile stages/leaflet) were similar at the time of the first sampling but in the second sampling they were higher in the presence of russet mites (Both mites) (Student’s t-test on log-transformed numbers; P = 0.73 and P <0.001, respectively). NS = not significant; the asterisk indicates a statistically significant difference. Russet mite densities (not shown in the figure) were similar at the time of the first sampling but were reduced in the presence of spider mites in the second sampling, albeit not significantly (Student’s t-test on log-transformed numbers; P = 0.89 and P = 0.09). Data are the same as those reported in Table S1. [file 12915_2014_98_MOESM2_ESM.pdf]

Supplemental text

Our field data give rise to three, not mutually exclusive, hypotheses on what determines spider mite and russet mite distribution patterns and their population sizes. First of all, we found co-occurrence of the two species at 7 of 85 sampling sites at the first sampling moment. In contrast, we found co-occurrence of the two species at 33 of 85 sites at the second sampling moment (Table S1): in 7 of these cases the order of infestation was unknown, but in 21 of the remaining 26 sites the spider mite-infestation had followed the russet mite-infestation (and hence, in only 5 of the 26 sampling sites the russet mite-infestation had followed the spider mite-infestation). These absence/presence field data suggest that spider mites may actively select plants pre-infested with russet mites and therefore succeed these more often than the other way around (the "succession hypothesis"). Second of all, our field data show that spider mite populations reach well over 2-fold higher densities on plants pre-infested with russet mites than on plants without russet mites (Figure S2). These population density field-data suggest that spider mites may be arrested to plants co-infested with russet mites longer than to plants without russet mites (the "arrestment hypothesis") and/or that spider mites have a higher intrinsic population growth-rate on russet mite-infested plants due to facilitation (the "facilitation hypothesis").

The *succession* hypothesis is questionable since spider mites are semi-passive dispersers, i.e. they do not actively select host plants but are dispersed by wind (Smitley & Kennedy, 1988). The same applies to russet mites (Sabelis & Bruin, 1996) but especially the dispersal behavior of spider mites has been documented in detail. Spider mites are known to migrate actively only within plants or between plants when these touch each other: usually they migrate towards uninfested leaves or away from natural enemies (Kennedy & Smitley, 1985; Pallini *et al.*, 1999). However, once arrived on an isolated plant, these mites can only decide whether to stay or try to depart again. Wind dispersal behavior is characterized by the mites positioning themselves at the edge of a leaf blade or the tip of a plant (where the boundary layer is thin) and this behavior can be induced by several (a)biotic factors, such as overexploitation, drought and/or the presence of predators (Smitley & Kennedy, 1988). Together, this strongly suggests that spider mites will not often actively select new (more favorable) host plants. However, finding spider mites more often on plants pre-infested with russet mites than the other way around can also be indicative of differential *arrestment* (i.e. in this scenario there is not active selection but both mites occasionally land on plants already infested by the other species by chance while spider mites have a lower tendency to disperse from such plants than russet mites). Yet, statistically evaluating either differential succession or differential arrestment is not straightforward since the expected values (probabilities) for such a

test can only be derived from the proportion of all plants (including the ones without mites) that were attacked by spider mites and the proportion of all plants that were attacked by russet mites prior to the second sampling. Since in our study the two sampling moments were 2 months apart it is doubtful if the first sampling can be used for calculating such expected values reliably. Finally, also phenological differences between the mites rather than their host preferences could give rise to an apparent difference in succession or arrestment. In Italy, russet mites often emerge somewhat earlier in the season on Solanaceae than spider mites (Castagnoli *et al*., 1998) but both mite species need 10-15 degrees Celsius to complete their life cycle and perform optimally at 25-30 ºC and both prefer dry conditions (Kawai & Haque, 2004; Crooker, 1985). Moreover, the intrinsic rate of natural increase of russet mites at 25 ºC is similar to that of spider mites (Kawai & Haque, 2004). Hence, their moment of occurrence and their average population growth-rate largely coincide and, consequently, both are able to complete around 8-10 generations per year. In addition, also the fact that we found spider mites on a similar number of locations during the first sampling (47) as during the second sampling (46) suggests there are no substantial phenological differences between the two species at play. Taken together, it is unlikely that spider mites select favorable host plants in the field and although it is possible that spider mites are arrested longer on russet mite-infested plants than on uninfested plants providing statistical support for this is challenging.

The *arrestment* hypothesis and the *facilitation* hypothesis are not mutually exclusive and difficult to entangle. Yet, the facilitation hypothesis was supported strongly by our laboratory experiments. Whether or not facilitation evokes also longer arrestment is unclear but our laboratory experiments show that indirect *plant-mediated facilitation* largely accounts for the observed differences in spider mite population-densities on field-grown tomatoes with and without russet mites.

Table S1: Spider mites and russet mites co-occur in the field.

	Location	Sampling 1	Sampling 1	Sampling 1	Sampling 2	Sampling 2	Sampling 2
		**SM alone (mites/ leaflet)**	**RM alone (mites/ leaflet)**	**Both mites (SM/RM per leaflet)**	**SM alone (mites/ leaflet)**	**RM alone (mites/ leaflet)**	**Both mites (SM/RM per leaflet)**
1	Oliva Aversana, Battipaglia, SA	1.6			5.6		
2	Oliva Aversana, Battipaglia, SA	1.5			4.1		
3	Vallecchi, Cannara, PG	2.1			7.1		
4	Bellandi G., S. Agata, Scarperia, FI	1.9			5.1		
5	Bellandi G., S. Agata, Scarperia, FI	2.8			3.3		
6	Fabbri R., S. Agata, Scarperia, FI	1.0			2.2		
7	Fabbri R., S. Agata, Scarperia, FI	1.0			4.4		
8	Fabbri L., S. Agata, Scarperia, FI	2.6			2.7		
9	Di Nola, Lucera, FG	2.3			3.5		
10	Oliva Aversana, Battipaglia, SA	1.5			7.9		
11	Oliva Aversana, Battipaglia, SA	2.0			14.0		
12	Vinciarelli, Cesa, AR	4.0			1.5		
13	Arsia, Cesa, AR	3.4			5.4		
14	Arsia, Cesa, AR	2.0			1.4		
15	Arsia, Cesa, AR	6.6			2.3		
16	Vinciarelli, Callone, Montepulciano, SI	6.3			1.6		
17	Vinciarelli, Callone, Montepulciano, SI	2.7			3.2		
18	Meini, Poggetti, GR	8.9			2.8		
19	Marioni, Marrucheto, GR	6.9			4.7		
20	Braccagni, Braccagni, GR	11.0			10.5		
21	Braccagni, Braccagni, GR	2.0			5.2		
22	Rurimoni, Montalto di Castro, VT	17.2			14.5		
23	Bartoccini, Canino, VT	3.0			4.5		
24	Bartoccini, Canino, VT	13.5			7.2		
25	Bartoccini, Canino, VT	4.7			5.1		
26	Vallepega, Comacchio, FE	3.9			2.1		
27	Vallepega, Comacchio, FE	7.3			5.8		
28	Vallepega, Comacchio, FE	16.5			21.2		
29	Vallepega, Comacchio, FE	16.3			9.9		
30	Di Nola, Lucera, FG	5.2			6.7		
31	Cooperativa le Rose, Impruneta, FI	4.6			9.7		
32	Cooperativa le Rose, Impruneta, FI	1.8			4.0		
33	Frolani, Bolgheri, LI	1.3			4.4		
34	Bernardini, Affitti, Piombino, LI	8.3			7.1		
35	Coccini, Cannara, PG	5.0			6.1		
36	Coccini, Cannara, PG	18.2			9.4		
37	Coccini, Cannara, PG	4.7			3.3		
38	Veneziani, S. Giorgio Piac., PC	10.5			13.8		
39	S. Nazarro di Sissa, PR	5.1			8.5		
40	Ragazzola di Roccabianca, PR	27.0			18.5		
41	Soragna, PR	3.5			2.4		
42	Calabrina, Cesena, FC	13.4			1.9		
43	Campo 4, Caorso, PC	2.2					28.9 / 12.5
44	Oliva, Aversana, Battipaglia, SA	3.4					46.6 / 23.9
45	Galassi R, Cannara, PG	1.0					15.1 / 46.1
46	Dragoni, Sterpeto, GR	1.0					10.3 / 58.7
47	Arsia, Rispescia, GR	2.2					9.1 / 18.3
48	Arsia, Cesa, AR				1.8		
49	Ragazzola, Ragazzola di Roccabianca, PR				2.1		
50	Vinciarelli, Cesa, AR				2.6		
51	Cooperativa le Rose, Impruneta, FI				3.5		

52	Arsia, Cesa, AR		32.5			51.9	
53	Vinciarelli, Callone, Montepulciano, SI		31.0			25.4	
54	Vinciarelli, Cesa, AR		23.8			29.7	
55	Vinciarelli, Cesa, AR		15.3			12.9	
56	Cooperativa le Rose, Impruneta, FI		22.8			46.4	
57	San Bonico, PC		135.4				7.4 / 25.3
58	Torre Mar., Montalto di Castro, VT		27.5				2.2 / 13.9
59	Valentini, S. Giorgio Piac., PC		62.9				10.5 / 10.9
60	Rurimoni, Montalto di Castro, VT		33.1				8.1 / 17.9
61	Saliceto di Cadeo, PC		22.5				16.1 / 8.9
62	Veneziani, S. Giorgio Piac., PC		8.0				4.8 / 1.9
63	Saliceto di Cadeo, PC		12.0				19.8 / 5.2
64	Monticello, Caorso, PC		18.0				3.1 / 3.4
65	Bondiocca di Caorso, PC		23.0				13.6 / 2.5
66	Casalfoschino di Sissa, PR		13.0				11.1 / 2.2
67	Trecasali, PR		24.5				21.3 / 11.4
68	Bianconese di Fontevivo, PR		18.0				3.9 / 4.2
69	Prati B. Calabrina, Cesena, FC		38.5				12.9 / 9.6
70	Capannaguzzo, Cesena, FC		18.5				15.0 / 8.0
71	Milano, Montalto di Castro, VT		24.0				18.0 / 3.8
72	Arsia, Cesa, AR		28.5				9.2 / 2.3
73	Meini, Poggetti, GR		13.5				7.0 / 1.3
74	Arsia, Rispescia, GR		23.4				11.0 / 11.4
75	Dragoni, Poggio al Pino, GR		28.7				3.7 / 8.9
76	Marioni, Poggio al Pino, GR		19.4				10.9 / 3.8
77	Bondiocca di Caorso, PC		11.8				13.9 / 4.2
78	Cooperativa le Rose, Impruneta, FI					3.8	
79	Meini, Vareop., GR			1.5 / 53.5			8.8 / 52.9
80	Vallepega, Comacchio, FE			2.5 / 13.8			7.7 / 14.0
81	Dragoni, Favacchio, GR			15.4 / 14.4			18.9 / 13.8
82	Gianandrea, Rosignano, LI			1.8 / 28.1			6.5 / 28.8
83	Di Francesco, La California, Bibbona, LI			4.8 / 11.7			17.9 / 12.8
84	Frolani, Bolgheri, LI			3.8 / 43.8			13.1 / 48.4
85	Di Nola, Lucera, FG			3.4 / 19.2			20.4 / 17.7

List of samplings documenting the (co-)occurrence of russet mites (RM) and spider mites (SM) in field-grown tomatoes in Italy. Spider mites and/or russet mites were found on tomato at 85 field sites. In 33 field sites they co-occurred on the same host plant. Numbers refer to mite densities (motile stages/leaflet). In the case of double infestations, SM density is reported first followed by the RM density. Locations are indicated by the name of the farm (if applicable), followed by the name of the town and region (SA=Salerno, PG=Perugia, FG=Foggia, LI=Livorno, GR=Grosseto, AR=Arezzo, VT=Viterbo, FE=Ferrara, PC=Piacenza, PR=Parma, FC=Forlì-Cesena, FI=Firenze, SI=Siena).

Figure S2

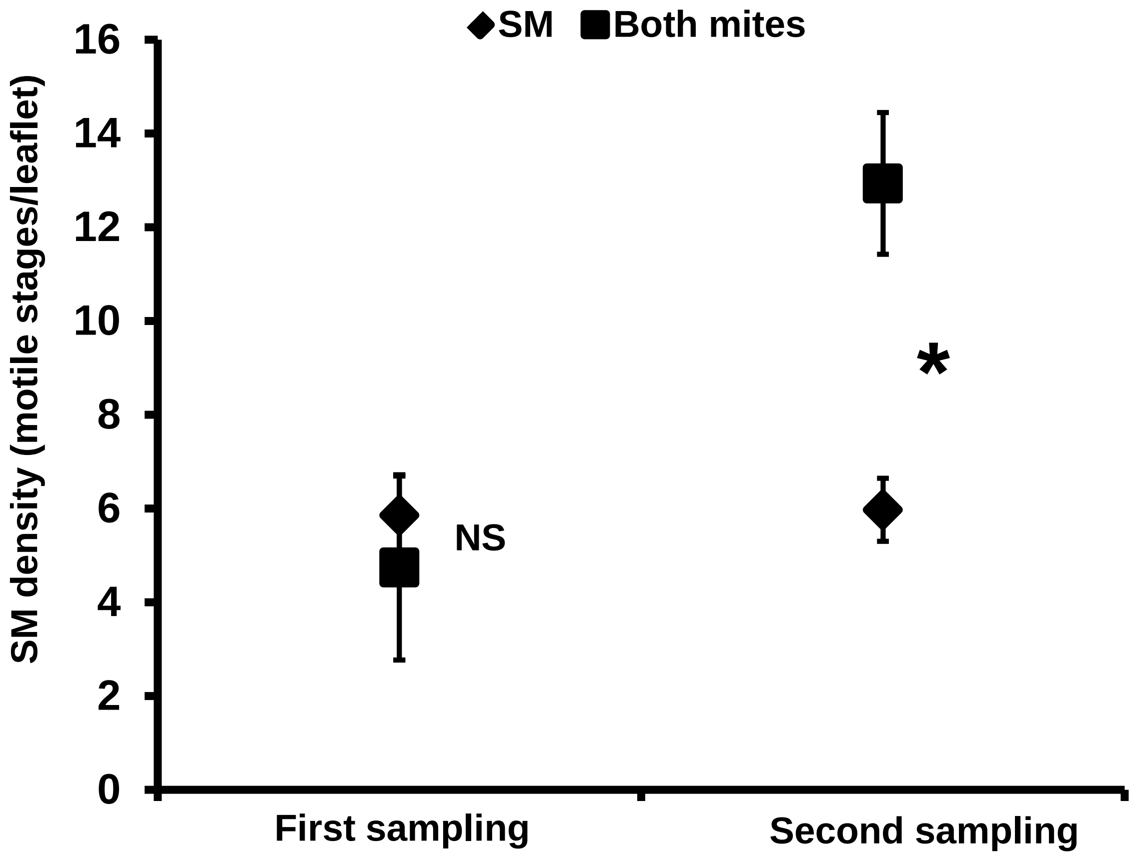
SM
Both mites
16
14
12
10
8
6
4
2
0
SM density (motile stages/leaflet)
NS
*
First sampling
Second sampling

Supplemental references

1. Castagnoli M, Liguori M, Nannelli R, Simoni S: **Preliminary survey on mite fauna of tomato in Italy.** *Redia* 1998, **81**:45-54 (in Italian with English abstract).

2. Crooker A: **Embryonic and juvenile development.** In: *Spider mites - their biology, natural enemies, and control*. Edited by Helle W, Sabelis MW. Vol. 1A. Amsterdam: Elsevier; 1985:149–163.

3. Kawai A, Haque MM: **Population dynamics of tomato russet mite, *Aculops lycopersici* (Massee) and its natural enemy, *Homeopronematus anconai* (Baker).** *JARQ* 2004, **38**:161-166.

4. Kennedy GG, Smitley DR: **Dispersal.** In: *Spider mites - their biology, natural enemies, and control*. Edited by Helle W, Sabelis MW. Vol. World Crop Pest Series 1A. Amsterdam: Elsevier; 1985:233–242.

5. Pallini A, Janssen A, Sabelis MW: **Spider mites avoid plants with predators.** *Exp Appl Acarol* 1999, 23:803-815.

6. Sabelis MW, Bruin J: **Evolutionary ecology: life history patterns, food plant choice and dispersal.** (1996) In: *Eriophyoid mites – their biology, natural enemies and control.* Edited by Lindquist EE, Sabelis MW, Bruin J. Vol. World Crop Pest Series 6. Amsterdam: Elsevier; 1996: 329-366.

7. Smitley DR, Kennedy GG: **Aerial dispersal of the two-spotted spider mite (*Tetranychus urticae*) from field corn.** *Exp Appl Acarol* 1988, **5**:33-46.
