# Supplementary figures and images for: Defense suppression benefits herbivores that have a monopoly on their feeding site but can backfire within natural communities
Source: BMC Biol. 2014 Nov 18;12:98. doi: 10.1186/s12915-014-0098-9 (PMC4258945; doi:10.1186/s12915-014-0098-9)

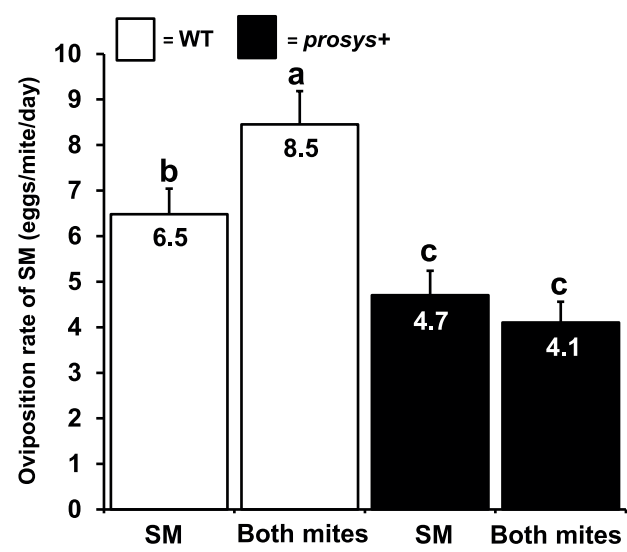

Supplement: Additional file 3: Figure S3. — Spider mite performance is not affected by the presence of russet mites on transgenic 35S::prosystemin tomatoes. Average number of eggs produced by spider mites (SM) on wild-type (cv. CM) and transgenic 35S::prosystemin plants (prosys+) that were pre-infested with RM (Both mites) or not (SM). Values in the bars (+SEM) represent means and different letters above bars indicate significant differences in SM oviposition rate (ANOVA followed by Fisher’s LSD test, P <0.05). Three leaflets per plant were analyzed. In total, 10 plants per genotype and per treatment were analyzed. [file 12915_2014_98_MOESM3_ESM.pdf]

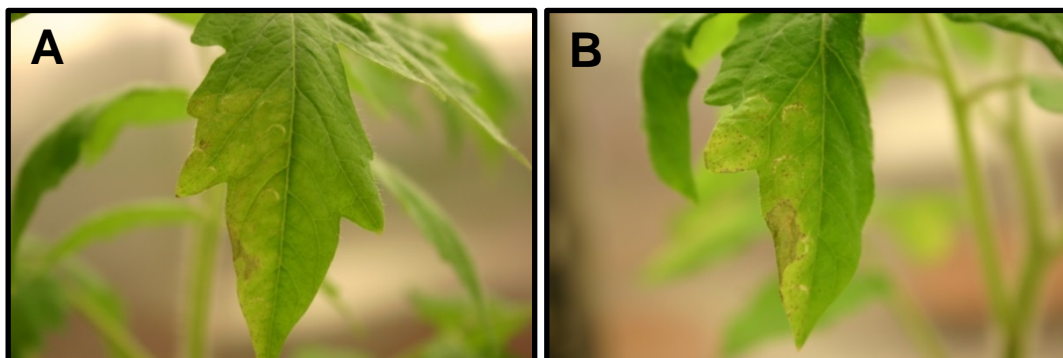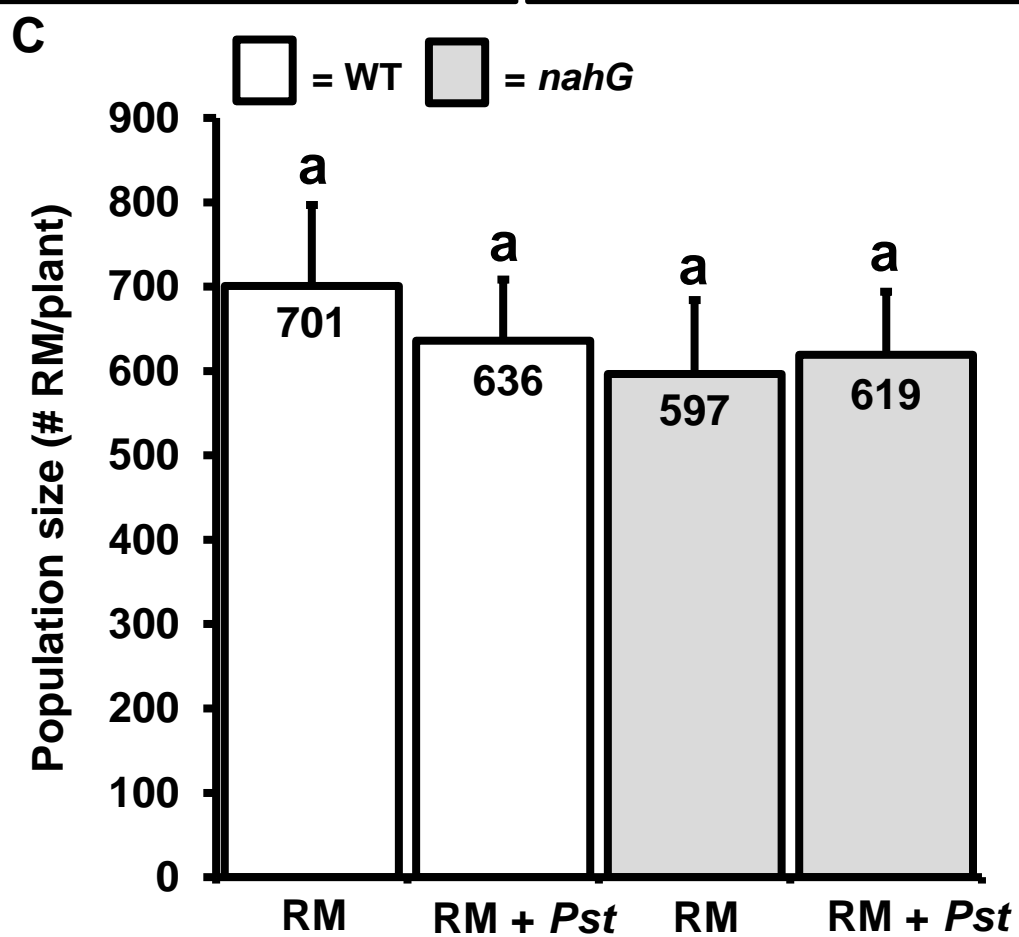

Supplement: Additional file 5: Figure S5. — Pst DC3000 infections do not interfere with russet mite population growth. Symptoms of bacterial infection on leaflets of wild-type (cv. MM) (A) and nahG (B) plants. Russet mite population size (number of mites/plant; + SE) on wild-type (cv. MM) and nahG plants infested with RM alone (RM) or co-infected with Pst (RM + Pst) 14 days after infestation with RM. Plants were infiltrated with Pst seven days after infestation with RM. Values represent the mean of 15 plants. Letters above bars indicate results according to ANOVA (P = 0.83). [file 12915_2014_98_MOESM5_ESM.pdf]

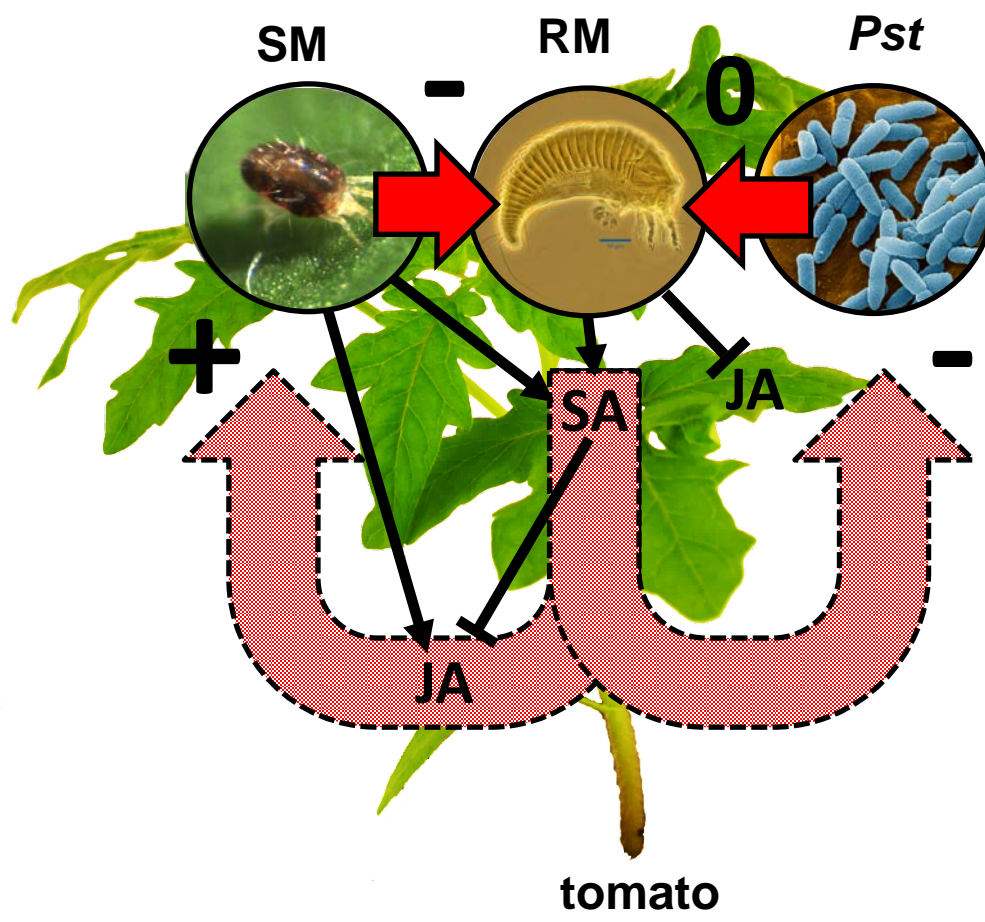

Supplement: Additional file 6: Figure S6. — Schematic diagram of the indirect interactions occurring between russet mites, spider mites and Pst DC3000. SM = spider mite; RM = russet mite; Pst = Pseudomonas syringae pv. tomato; JA = jasmonic acid; SA = salicylic acid. [file 12915_2014_98_MOESM6_ESM.pdf]

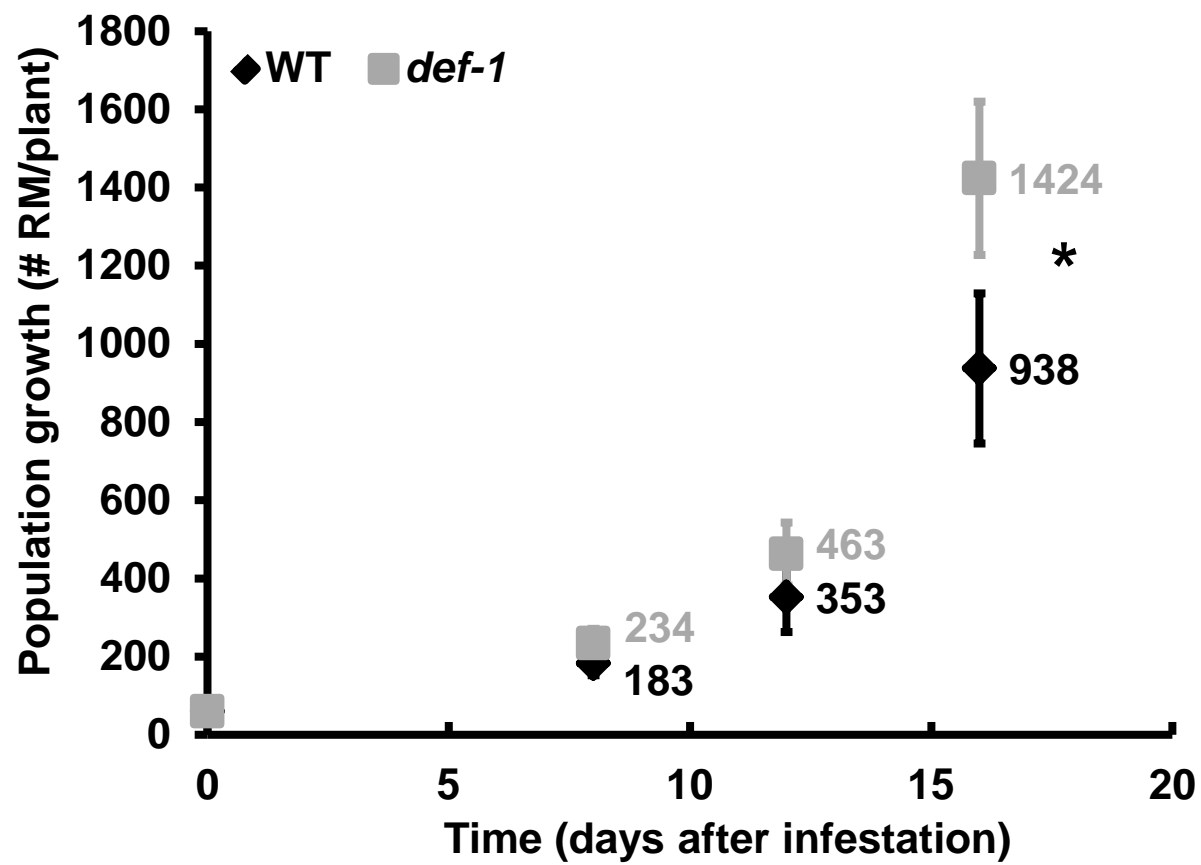

Supplement: Additional file 7: Figure S7. — Russet mites grow faster on the JA-deficient mutant def-1 than on wild-type (WT) plants. Values (±SE) represent the mean number of russet mites (RM) per plant, obtained from five to ten plants in two independent experiments. The asterisk indicates a significant difference after 16 days (Student’s t-test on log-transformed data; P = 0.036). [file 12915_2014_98_MOESM7_ESM.pdf]

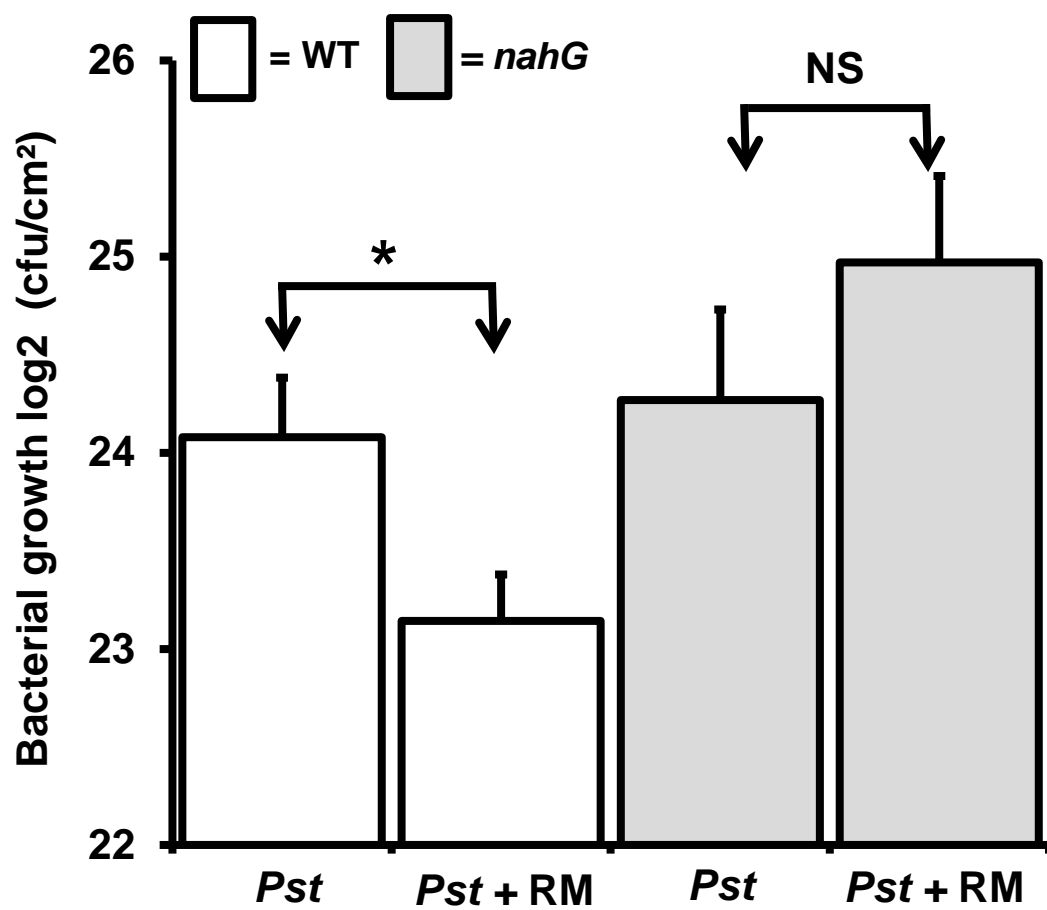

Supplement: Additional file 9: Figure S8. — Repetition of the Pst DC3000 growth experiment. Pst population growth, quantified as the number of colony forming units (CFU/cm2; + SE), in wild-type (WT) (cv. MM) and nahG plants that were either without RM (Pst) or had been pre-infested with RM (Pst + RM) for seven days. In total, six plants were used per genotype and per treatment. The asterisk indicates a statistically significant difference (Student’s t-test; P <0.05). [file 12915_2014_98_MOESM9_ESM.pdf]

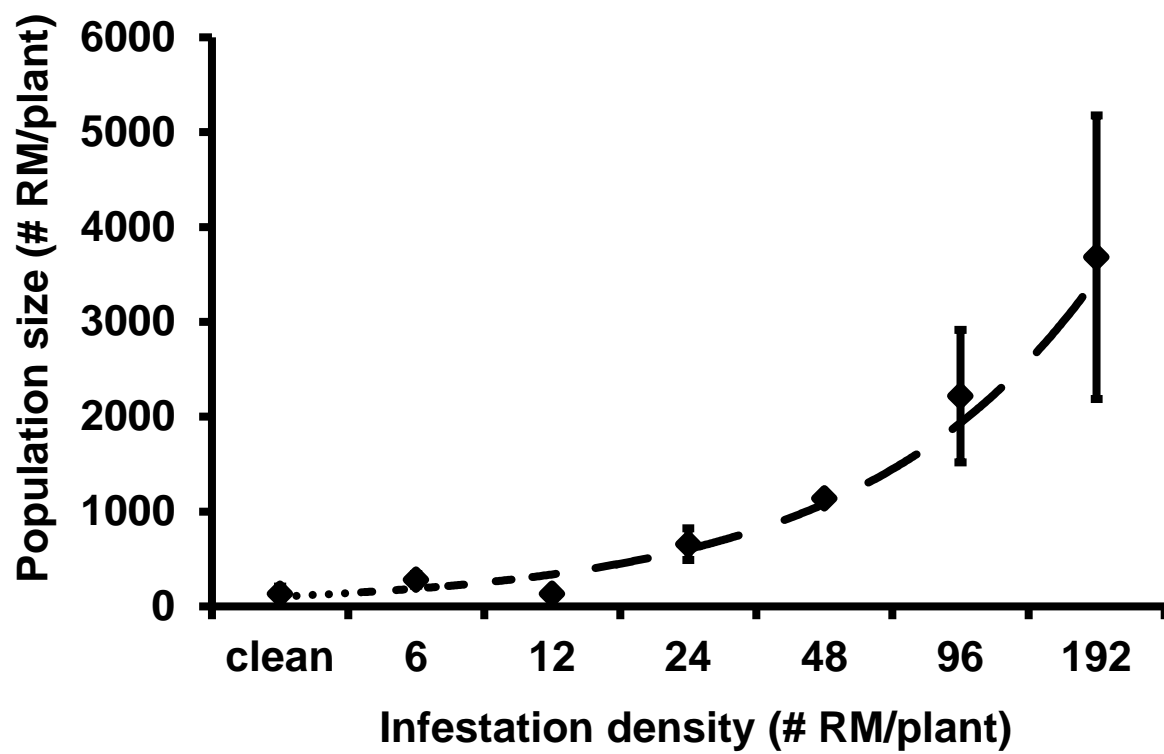

Supplement: Additional file 10: Figure S9. — Dose–response experiment. Values (±SE) represent the mean number of russet mites (RM) per plant 14 days after infestation. In total, two plants per density were analyzed. [file 12915_2014_98_MOESM10_ESM.pdf]
