# Supplementary material for: Defense suppression benefits herbivores that have a monopoly on their feeding site but can backfire within natural communities
Source: BMC Biol. 2014 Nov 18;12:98. doi: 10.1186/s12915-014-0098-9 (PMC4258945; doi:10.1186/s12915-014-0098-9)
Supplement: Additional file 4: Figure S4. — Pest distribution data for russet mites (Aculops lycopersici), spider mites (Tetranychus urticae) and the bacterial pathogen Pseudomonas syringae pv. tomato (source: [54], accessed on 24/09/2014 and [55]). [file 12915_2014_98_MOESM4_ESM.pdf]

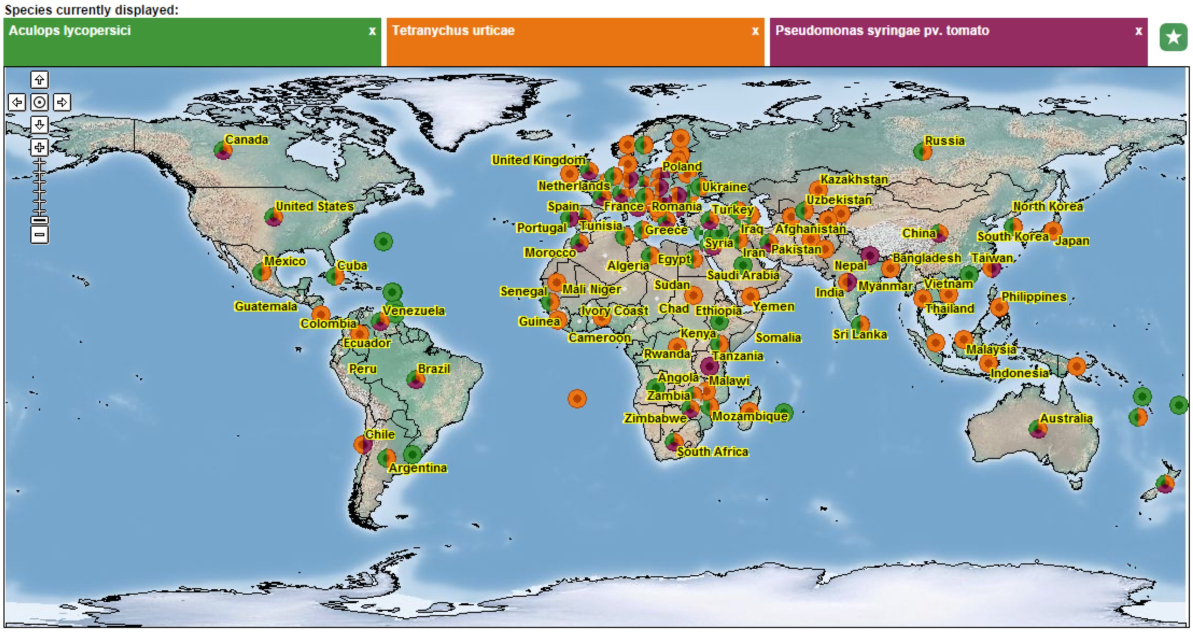

Country level records, 1 species

Species 1

Species 2

Species 3

Country level records, 2 species

Species 1 & 2

Species 1 & 3

Species 2 & 3

Country level, species 1, 2 & 3
