# Supplementary material for: Defense suppression benefits herbivores that have a monopoly on their feeding site but can backfire within natural communities
Source: BMC Biol. 2014 Nov 18;12:98. doi: 10.1186/s12915-014-0098-9 (PMC4258945; doi:10.1186/s12915-014-0098-9)
Supplement: Additional file 8: Table S2. — Accession numbers of the transcripts quantified by qRT-PCR and their primer sequences. [file 12915_2014_98_MOESM8_ESM.pdf]

Table S2: Accession numbers of the transcripts quantified by qRT-PCR and their primer sequences.

**Target Gene**	**GenBank (GB) ID**	**GenModel (ITAG2.3)**	**Primer Sequence**
ACT	XM_004235020.1	Solyc03g078400.2	QF 5’-TTAGCACCTTCCAGCAGATGT-3’<br>QR 5’-AACAGACAGGACACTCGCACT-3’
PPO-F	AK247126.1	Solyc08g074630.1.1	QF 5’-CGGAGTTTGCAGGGAGTTATAC-3’<br>QR 5’-TTGATCTCCACACTTTCAATGG-3’
TD-II	M61915.1	Solyc09g008670.2	QF 5’-TGCCGTTAAAAATGTCACCA-3’<br>QR 5’- ACTGGCGATGCCAAAATATC-3’
JIP-21	AJ295638.1	Solyc03g098790.1	QF 5’-ACTCGTCCTGTGCTTTGTCC-3’<br>QR 5’-CCCAAGAGGATTTTCGTTGA-3’
WIPI-II	AY129402.1	Solyc03g020080.2	QF 5’-GACAAGGTACTAGTAATCAATTATCC-3’<br>QR 5’-GGGCATATCCCGAACCCAAGA-3’
PR-P6	M69248.1	Solyc00g174340.1	QF 5’-GTACTGCATCTTCTTGTTTCCA-3’<br>QR 5’-TAGATAAGTGCTTGATGTCCA-3’
