# Supplementary material for: Defense suppression benefits herbivores that have a monopoly on their feeding site but can backfire within natural communities
Source: BMC Biol. 2014 Nov 18;12:98. doi: 10.1186/s12915-014-0098-9 (PMC4258945; doi:10.1186/s12915-014-0098-9)
Supplement: Additional file 1: Figure S1. — Map of Italy with the regions indicated where russet mites and spider mites were found. The color of the symbols indicate which species were found (russet mites = yellow; spider mites = green; both mites = red). [file 12915_2014_98_MOESM1_ESM.pdf]

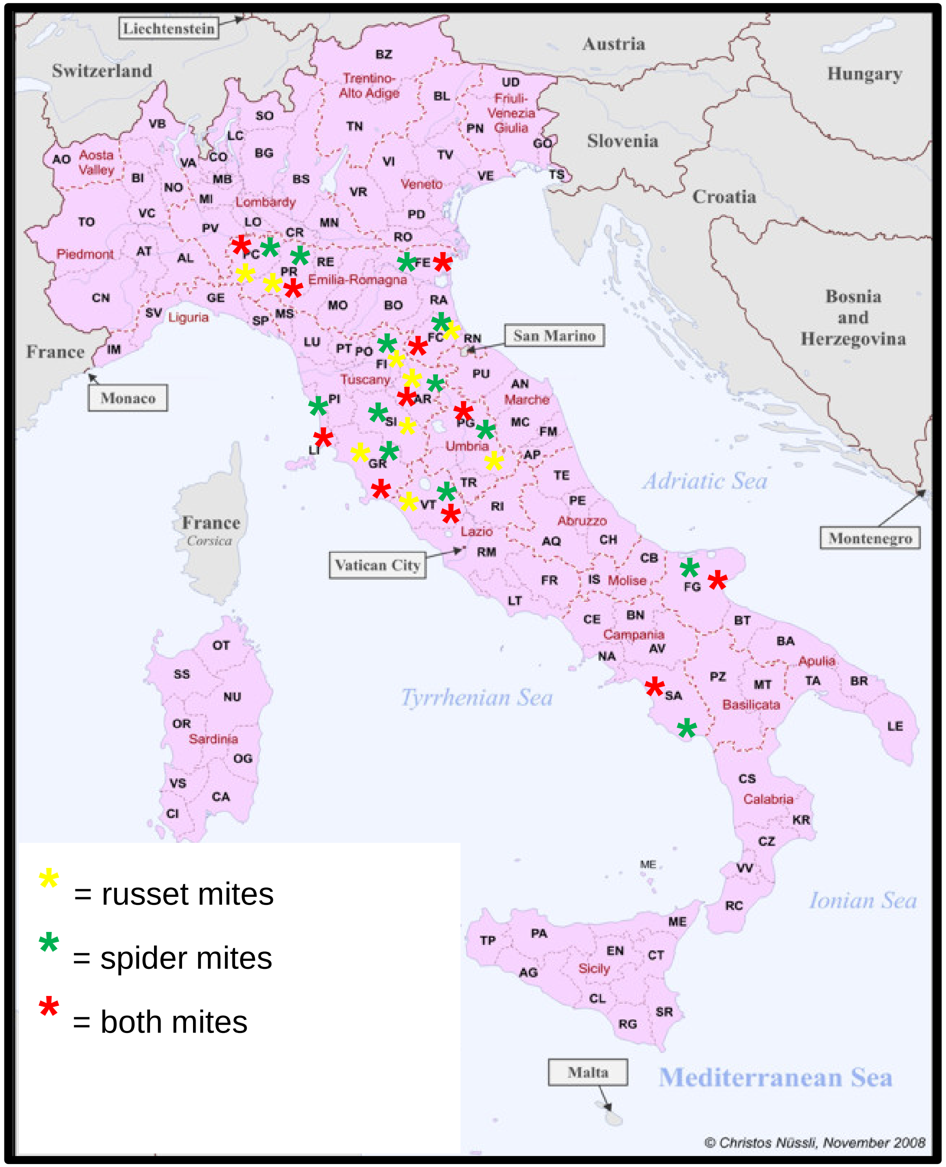
Liechtenstein
Switzerland
Austria
Hungary
Slovenia
Croatia
Bosnia and Herzegovina
France
Monaco
San Marino
Vatican City
Montenegro
Malta
France
Corsica
Adriatic Sea
Tyrrhenian Sea
Ionian Sea
Mediterranean Sea
Aosta Valley
Piedmont
Liguria
Lombardy
Trentino-Alto Adige
Friuli-Venezia Giulia
Veneto
Emilia-Romagna
Tuscany
Marche
Umbria
Lazio
Abruzzo
Molise
Campania
Apulia
Basilicata
Calabria
Sicily
Sardinia
AO
VB
BI
NO
VC
TO
AT
AL
CN
SV
IM
GE
SP
VA
CO
LC
SO
MB
MI
BG
BS
LO
PV
CR
MN
PC
PR
RE
MO
BO
FE
RA
FC
RN
MS
LU
PT
PO
FI
PI
LI
SI
AR
GR
BZ
TN
BL
UD
PN
GO
TS
VI
TV
VR
PD
VE
RO
PU
AN
MC
FM
AP
PG
TR
VT
RI
RM
LT
FR
TE
PE
AQ
CH
CB
IS
FG
BT
BA
TA
BR
LE
CE
BN
NA
AV
SA
PZ
MT
CS
KR
CZ
VV
RC
ME
TP
PA
AG
EN
CT
CL
RG
SR
OT
SS
NU
OR
OG
VS
CA
CI
* = russet mites
* = spider mites
* = both mites
© Christos Nüssli, November 2008
